# Separating Lentiviral Vector Injection and Induction of Gene Expression in Time, Does Not Prevent an Immune Response to rtTA in Rats

**DOI:** 10.1371/journal.pone.0009974

**Published:** 2010-04-01

**Authors:** David M. Markusic, Dirk R. de Waart, Jurgen Seppen

**Affiliations:** Academic Medical Center, Tytgat Institute for Liver and Intestinal Research, Amsterdam, The Netherlands; Centre de Recherche Public de la Santé (CRP-Santé), Luxembourg

## Abstract

**Background:**

Lentiviral gene transfer can provide long-term expression of therapeutic genes such as erythropoietin. Because overexpression of erythropoietin can be toxic, regulated expression is needed. Doxycycline inducible vectors can regulate expression of therapeutic transgenes efficiently. However, because they express an immunogenic transactivator (rtTA), their utility for gene therapy is limited. In addition to immunogenic proteins that are expressed from inducible vectors, injection of the vector itself is likely to elicit an immune response because viral capsid proteins will induce “danger signals” that trigger an innate response and recruit inflammatory cells.

**Methodology and Principal Findings:**

We have developed an autoregulatory lentiviral vector in which basal expression of rtTA is very low. This enabled us to temporally separate the injection of virus and the expression of the therapeutic gene and rtTA. Wistar rats were injected with an autoregulatory rat erythropoietin expression vector. Two or six weeks after injection, erythropoietin expression was induced by doxycycline. This resulted in an increase of the hematocrit, irrespective of the timing of the induction. However, most rats only responded once to doxycycline administration. Antibodies against rtTA were detected in the early and late induction groups.

**Conclusions:**

Our results suggest that, even when viral vector capsid proteins have disappeared, expression of foreign proteins in muscle will lead to an immune response.

## Introduction

Many forms of gene therapy will require the ability to modulate the expression of therapeutic genes to maintain expression levels within a therapeutic window or adjust expression levels based on disease progression within the patient [1]. Lentiviral vectors derived from HIV-1 are a well-suited vehicle for the treatment of a variety of inherited and acquired diseases. They can deliver a relatively large therapeutic cassette into both dividing and non-dividing cells and integrate into the host cell genome providing life long expression of the therapeutic gene [2]–[4].

Because the tetracycline (Tet-On) inducible system [5] has been extensively used to regulate gene expression *in vitro* and *in vivo* [6]–[8], it is an attractive candidate to develop regulated gene therapy. The Tet-On system is composed of a chimeric reverse tetracycline transactivator (rtTA) composed of the herpesvirus VP16 transcription activating domain and a mutant tetracycline repressor protein from *Escherichia coli.* Furthermore, the system contains a minimal CMV promoter fused to several copies of the tetracycline operator sequence (tetO). In the presence of tetracycline or doxycycline, rtTA binds to the tetO and thus initiates transcription.

Early versions of the Tet-On system required high concentration of doxycycline for activation (100 ng/ml to 1000 ng/ml), which are easily obtained in cell culture but not *in vivo*. Novel rtTA variants derived from viral evolution were recently described that are responsive to as little as 10 ng/ml doxycycline [9], making them potentially better suited for *in vivo* use.

Although there have been significant improvements made in the basal activity and sensitivity of rtTA, this chimeric bacterial and viral protein can be a potent immunogen. Indeed, in studies performed in mice [10] and non-human primates [11], [12], the development of rtTA antibodies and cytotoxic T cell mediated clearance of rtTA expressing cells was observed.

Immune responses to therapeutic proteins and clearance of corrected cells is a major obstacle to the clinical implementation of gene therapy. The Danger Model proposed by Matzinger suggests that the immune system does not function solely based on detection of self and non-self, but additionally requires a danger signal to activate antigen presenting cells (APC) leading to an immune response [13], [14]. The Danger Model predicts that presentation of the antigen in the absence of danger signals would lead to either elimination or anergization of T cells and induce a temporary state of tolerance. In regards to gene therapy, the injection of a viral vector introduces large amounts of foreign proteins and is a potent trigger for the activation of danger signals [15].

We have previously described a single Tet-on inducible lentiviral vector with autoregulatory expression of rtTA [16]. This vector is characterised by very low basal expression levels of rtTA in the absence of doxycycline stimulation. Only when doxycycline is administrated, expression of rtTA and the therapeutic or marker gene is induced[16].

Mathematical modelling predicts that this type of synthetic gene circuit exhibits bimodal expression; the regulated gene can only be in a “on” or “off” state, without intermediary expression levels, and this was indeed verified experimentally[17]. However, the absolute magnitude of expression levels will vary between individual integrated vector genomes[17].

We showed in cell culture that our autoregulatory vector has lower background and higher induction than vectors in which there is constitutive expression of rtTA[16]. This vector also performed better *in vivo* in immunocompromised mice. Human hematopoietic stem cells were transduced with an autoregulatory or constitutive rtTA vector and transplanted into immune deficient mice[18]. Only cells transduced with the autoregulatory vector differentiated into multiple lineages and several cycles of GFP expression could be induced by doxycycline administration[18].

These data indicate that autoregulatory lentiviral vectors perform better than vectors in which there is constitutive expression of rtTA. Likely because constitutive expression of rtTA is toxic and also leads to higher background expression of the regulated gene.

We have previously shown that lentiviral vectors can be used for the stable long term expression of erythropoietin (Epo) in rats [19]. Erythropoietin gene therapy would be an alternative to treatment with recombinant Epo in patients with kidney failure. However, because over expression of Epo and the resulting high hematocrits will lead to a variety of clinical problems, Epo gene therapy must be carefully regulated.

To test our autoregulatory lentiviral vector system with a therapeutic gene in immunocompetent animals, we constructed an inducible rat Epo vector. This allowed us to determine if induction of rtTA expression in absence of the danger signals associated with the injection of viral particles, would avoid an immune response to rtTA and allow for repeated rounds of doxycycline stimulation.

## Results

### Construction and in vitro evaluation of TREAutoR4rEPO

**Figure 1:**
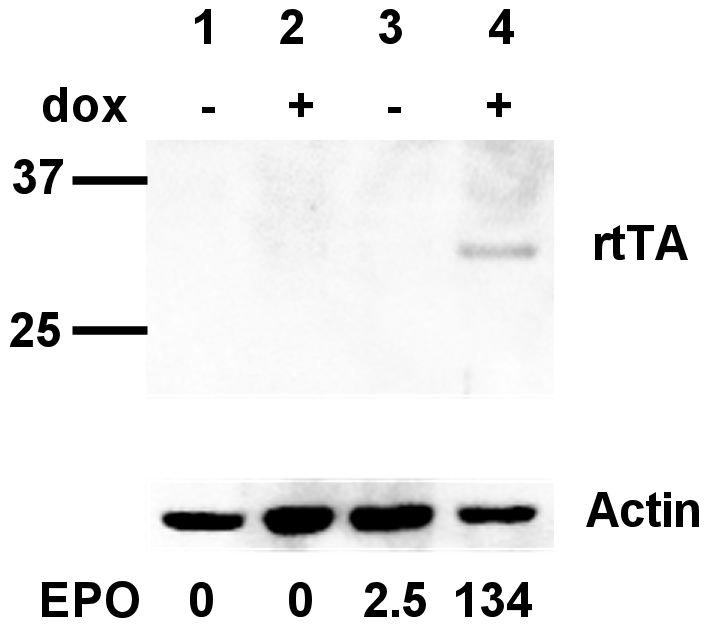
*In vitro* regulation of Epo expression in H9c2 rat myoblasts. A Western blot on control and transduced H9c2 cells was performed to detect rtTA expression. Expression of actin was used as a loading control. Simultaneously, the corresponding levels of Epo secreted into the cell culture media were measured by ELISA and are reported as mIU/mL below the Western blot. Lanes 1 and 2 control H9c2 lysates, 3 and 4 H9c2 cells transduced with TREAutoR4rEPO. Lanes 2 and 4 contain lysates of cells that were induced by the addition of doxycycline.

Our previously described single doxycycline inducible lentiviral vector TREAutoR3 [16] was modified by replacing the d2eGFP gene with the cDNA coding for rat erythropoietin and introduction of a novel rtTA variant, V16 (V9I F67S R171K) [9] with a 10 fold increased sentivity to doxycycline to make TREAutoR4rEPO. We selected a rat muscle cell line, H9C2, to evaluate the function of the system *in vitro*. H9C2 cells were transduced with concentrated TREAutoR4rEPO virus and allowed to expand. Following expansion, cells were stimulated with 10 ng/ml doxycycline and 72 hours later media was collected and cell lysates were prepared to determine basal and induced EPO and rtTA expression levels respectively. Western blot of H9C2 cell lysates showed undetectable levels of rtTA expression in the absence of doxycycline (Figure 1 lane 3), rtTA expression was only detected following treatment with 10 ng/ml doxycycline (Figure 1 lane 4). Control cells showed no detectable rtTA expression in the absence or presence of doxycycline (Figure 1 lanes 1). Epo was not detected in the media of mock transduced cells. (Figure 1 lower panel lanes 1 and 2). A low basal level of Epo was detected in the media of unstimulated cells transduced with the TREAutoR4rEPO vector (Figure 1 lower panel lane 3). Following stimulation with 10 ng/ml doxycycline we detected an strong increase in Epo levels (Figure 1 lower panel lanes 4) of 53 fold.

### Constitutive expression of rat Epo

**Figure 2:**
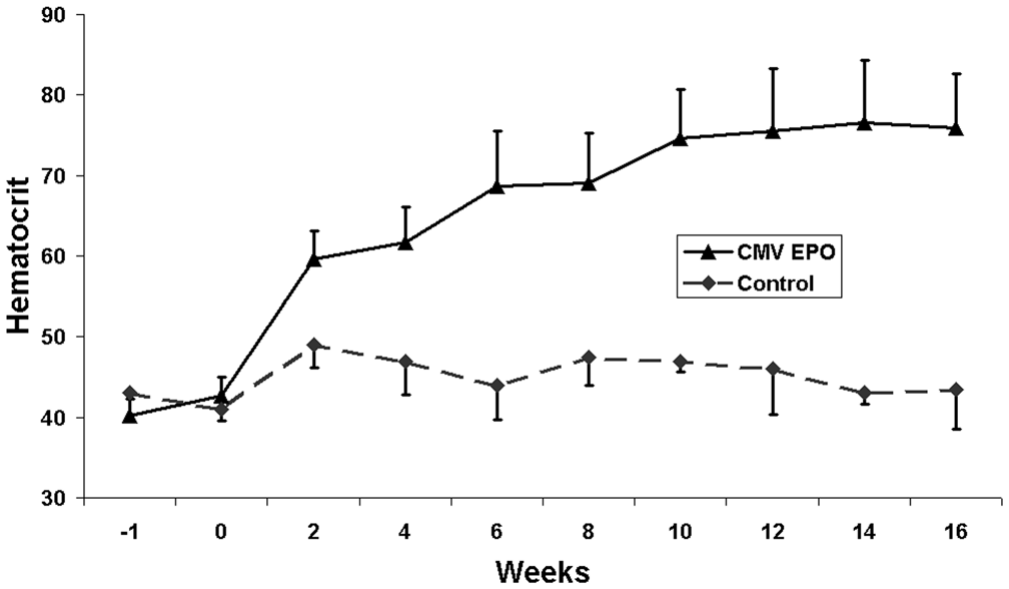
Constitutive lentiviral expression of Epo. Wistar rats were injected with CMV Epo (n = 11) or vehicle (n = 2) at t = 0. A sustained elevated hematocrit was observed in all CMV Epo injected animals.

We previously demonstrated long term expression of Epo delivered by an intramuscular injection of a lentiviral vector in Fisher 344 rats [19]. To confirm similar long-term expression in Wistar rats, a lentiviral vector with a constitutive CMV promoter driving expression of Epo was injected into the hind leg of Wistar rats and blood samples were collected every two weeks. An increase in hematocrit was observed two weeks following virus injection to 60%, reaching a plateau level of approximately 75% by week ten (Figure 2), confirming long term Epo expression. These data indicate that lentiviral vectors with constitutive expression of rat Epo are not silenced or cleared by the immune system in rat muscle.

### Inducible expression or rat Epo

**Figure 3:**
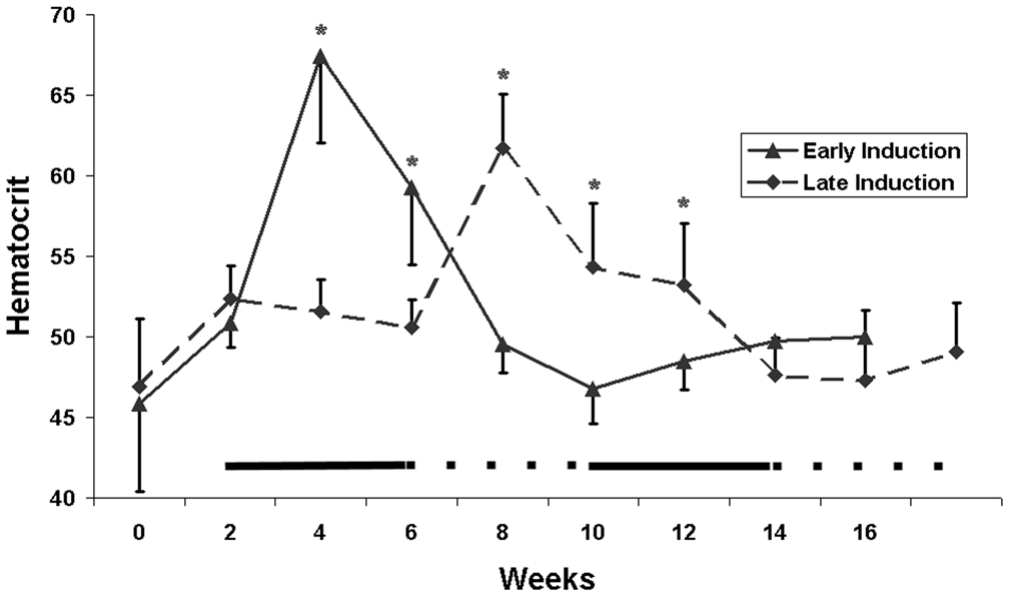
Inducible lentiviral expression of Epo. Wistar rats were injected with TREAutoR4rEpo at t = 0. Early induction rats (n = 7) were given doxycycline in the drinking water for two periods of four weeks starting at weeks 2 and 10 (solid line). Late induction rats (n = 5) were given doxycycline in the drinking water for two periods of four weeks starting at weeks 6 and 14 (dashed line). The solid and dashed lines below indicate the starting points for doxycycline administration for the early and late induction groups respectively. * Indicates a statistical significant difference p<0.05 between the early and late induction groups.

In total 16 rats received an IM injection of 0.4ug p24 of TREAutoR4rEPO virus in 100 µl PBS into the right hind leg. The animals were divided into two groups: early induction, receiving doxycycline in the drinking water two weeks following virus injection, and late induction getting doxycycline in the drinking water six weeks following virus injection. One rat from the early induction group and two from the late induction group did not have any response to doxycycline stimulation and were classified as non-responders. Blood was collected every two weeks for hematocrit determination. Two weeks following doxycycline administration the average hematocrit of the early induction group increased from 51% to 67% (Figure 3). Unexpectedly, after four weeks of doxycycline treatment the average hematocrit dropped down to 59%. Since the mean life span of erythrocytes in the Wistar rat is approximately 60 days [20], this rapid decrease in hematocrit suggests that there was a loss of Epo expression shortly after initiation of doxycycline administration. At the four and six week time points there was a significant difference (p<0.005) in hematocrit between untreated and doxycycline treated rats. A second round of stimulation with doxycycline was performed ten weeks following virus injection and no change in hematocrit was detected in all rats in the early induction group.

At six weeks following virus injection the early induction group was placed on normal water while the late induction group received its first doxycycline in the drinking water for a period of four weeks. Two weeks following doxycycline stimulation we observed an increase in hematocrit in the late induction group to approximately 62% (p<0.005) compared to virus injected rats on normal water. Because the magnitude in increase in hematocrit of the early and late induction groups were comparable, this demonstrated that transduced, non-induced cells persist for six weeks without being eliminated or silenced. As with the early induction group, we observed a rapid decrease in hematocrit after four weeks treatment with doxycycline, again suggesting a rapid elimination of transduced cells upon induction of Epo expression. A second round of doxycycline stimulation at week 14 failed to raise hematocrit levels as observed with the early induction group (Figure 3).

**Figure 4:**
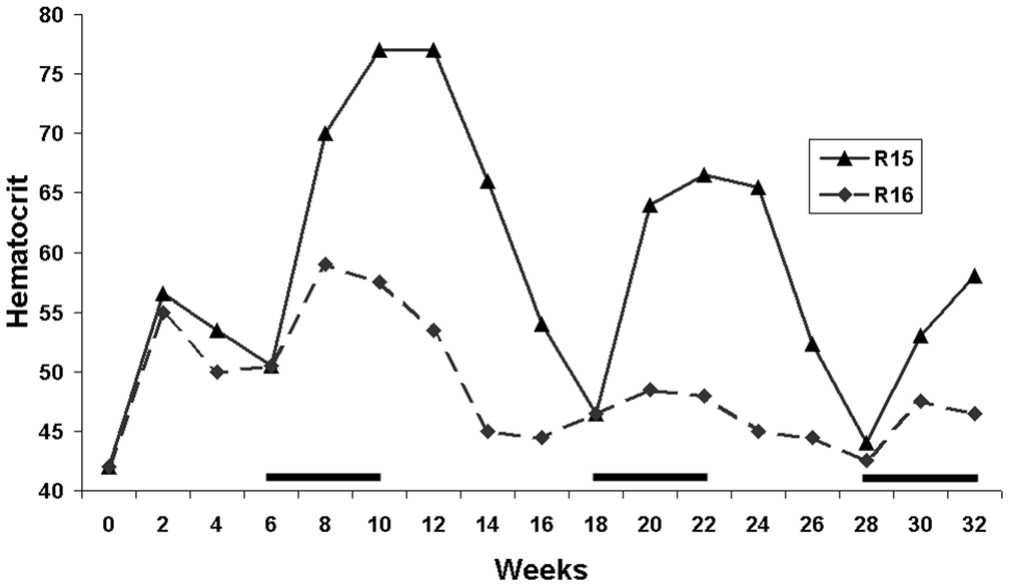
Multiple cycles of Epo expression. Wistar rats were injected with TREAutoR4rEpo at t = 0. Rats were given doxycycline in the drinking water for three periods of four weeks starting at weeks 6, 18 and 28 (solid line). In one rat (R15), hematocrits could be increased with each of three successive rounds of doxycycline administration. The cage and litter mate (R16) only responded once to doxycycline.

Interestingly, one rat, R15, within the late induction group was capable of undergoing multiple rounds of induction with doxycycline (Figure 4) and was not included in the data analysis for the late induction group. The kinetics of hematocrit changes of this rat were also markedly different. High hematocrits were maintained longer after doxycycline administration was halted. These observations provide more evidence that the rapid decrease in hematocrits in most animals induced by doxycycline was due to elimination of transduced cells.

### Detection of rtTA antibodies in plasma is independent of early or late induction

Plasma from rats at two, six, and ten weeks after viral injection was analyzed for the presence of rtTA antibodies in the early induction, late induction, and non-responder groups. No antibodies were detected in the plasma of animals from each group at the two weeks time point. At the ten week time point no difference was seen in the total number of animals positive for rtTA antibodies between the early and late induction groups (Table 1). This suggests that there is no benefit in delaying induction for preventing a humoral immune response.

**Table 1:** Antibodies directed against rtTA.

	Weeks following virus administration		
Group	2	6	10
Early Induction (n = 3)	0/3	2/3	3/3
Late Induction (n = 3)	0/3	1/3	3/3
Non responders (n = 2)	0/2	2/2	2/2

Eight animals were analyzed for the presence of circulating antibodies to rtTA by ELISA.

At 2 weeks following virus administration no animals had received doxycycline. At 6 weeks the Early induction group had been given doxycycline in the drinking water for 4 weeks and were subsequently placed on normal water. At 10 weeks the Late induction group had been given doxycycline in the drinking water for 4 weeks. The Non responders consist of one animal each from the Early and Late induction groups.

All animals developed antibodies to rtTA.

### Influx of immune cells detected within the area of viral injection

**Figure 5:**
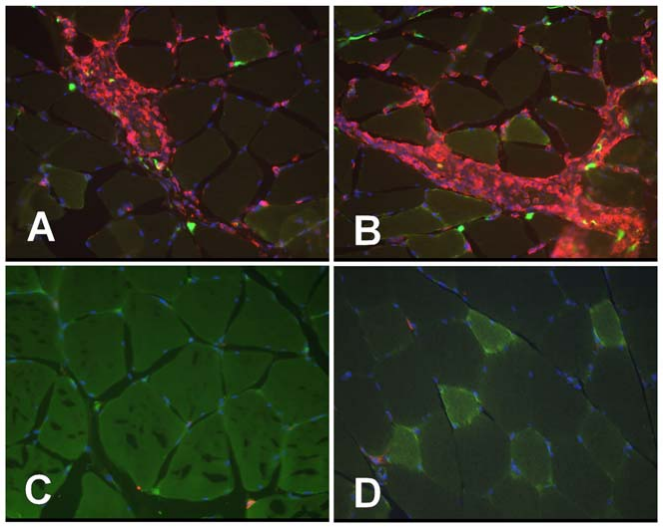
Histochemistry of rat muscle injected with GFP lentiviral vector. Wistar rats were injected with a lentiviral GFP expression vector (A-C) or saline control (D). (A,B) Areas with remnants of GFP expressing cells (green) show co-localization with CD45 stained (red) infiltrating immune cells. (C) Areas without GFP expression within the same piece of tissue are devoid of infiltrating immune cells. (D) Control injected muscle is negative for both GFP expression and infiltrating immune cells.

Rats were injected with a lentiviral GFP vector to better understand the local immunological response to an intramuscular injection. Animals were sacrificed at one and two weeks following injection and muscle was collected for immunostaining with an antibody directed against rat CD45 to detect the presence of infiltrating immune cells. One week following lentiviral vector injection we observed massive infiltration of CD45 positive cells in areas with remnants of GFP expression. (Figure 5A,B) and not in areas negative for GFP expression (Figure 5C) or in the PBS injected muscle (Figure 5D). Only punctuate GFP expression was detected, these are likely remnants of GFP expressing muscle cells. The muscle tissue with immune cell infiltration appeared damaged, also suggesting the clearance of transduced cells. No GFP positive muscle cells were detected two weeks following viral injection (data not shown). These data indicate that rats do mount a vigorous immune response to immunogenic antigens delivered by lentiviral vectors.

## Discussion

The ability to regulate the expression level of a therapeutic gene is vital for the advancement of gene therapy into the clinic. In addition to concerns over safety, expression levels of the therapeutic gene may require modulation in response to the disease progression of the patient[1]. Lentiviral vector mediated gene therapy is well suited for the long-term expression of a therapeutic gene. We have previously described an autoregulatory lentiviral vector that uses the tetracycline inducible system and is characterised by low basal expression of rtTA and regulated genes in the absence of doxycycline stimulation [16]. To test our system with a therapeutically relevant gene *in vivo* we constructed a vector in which Erythropoietin (Epo) expression could be regulated. This vector also included a new rtTA variant [9] which is 100 times more sensitive to doxycycline than the originally described rtTA.

We confirmed that by using this vector rtTA protein expression was undetectable on western blot of transduced muscle cells in the absence of doxycycline (Figure 1). We also showed that this vector mediates a robust induction of the therapeutic gene Epo *in vitro*.

Wistar rats were injected with the inducible Epo vector and doxycycline was administrated two weeks (early induction) or six weeks (late induction) after vector injection.

In both early and late and induction groups we observed a similar pattern, an initial increase in hematocrit at two weeks following doxycycline treatment followed by a reduction in hematocrit levels at four weeks on doxycycline (Figure 3). Given that the estimated life span of Wistar rat erythrocytes is 59 days [20], this suggests that immediately following doxycycline administration there is a rapid loss in exogenously expressed Epo. Silencing of vector is unlikely since rats injected with a constitutive Epo vector show long term expression without reduction in therapeutic effect (Figure 2) and because rats can be induced as efficiently two or six weeks after vector injection. Furthermore, in human hematopoietic cells grafted in immune deficient mice, multiple rounds of induction with a comparable autoregulatory GFP vector are also possible[18].

The most likely explanation of the loss of Epo expressing cells is therefore an cytotoxic immune response against the transduced muscle cells. This hypothesis is strengthened by the appearance of antibodies to rtTA in the injected animals. However, only direct detection of rtTA specific cytotoxic T cells will conclusively prove that the loss of Epo expression is immune mediated.

Interestingly, one animal in the late induction group was capable of undergoing multiple inductions (Figure 4), while the other animals were not responsive to a second round of doxycycline stimulation (Figure 3). Whether this was a consequence of the late induction is unclear and will require further investigations. No difference was observed in the development of antibodies against rtTA between non-responders, early induction, and late induction groups at ten weeks following virus injection (Table 1). This suggests that delaying induction has no benefit for the prevention of a humoral immune response.

A strong immune response was observed following intramuscular injected lentiviral vector expressing GFP. Muscle harvested one week following vector injection showed sporadic expression of GFP that was accompanied by massive infiltration of immune cells (Figure 5A and 5B). No GFP positive cells or areas of infiltrating immune cells were detected in an animal sacrificed two weeks following vector injection (data not shown), further suggesting a rapid clearance of GFP expressing muscle cells. These data show that rats have a vigorous immune response to lentiviral vectors expressing GFP from a ubiquitous promoter and might be a better animal model for pre clinical gene therapy studies than the commonly used murine models.

Several factors, including contaminants from the concentration of viral preparation [21] and efficient transduction of professional antigen presenting cells (APC) [22], [23], may play a role in the short term expression observed following intramuscular injection of lentiviral vectors. Introduction of muscle specific promoters have been shown to improve long term adenoviral [24] and AAV [25] mediated expression. A muscle specific promoter driving rtTA expression was required to obtain long term regulation using an adenoviral vector [26]. Together, this suggests that gene transfer to antigen presenting cells within the muscle and subsequent expression and presentation of the transgene may be a critical factor in the initiation of an immune response.

In summary, we have shown that the low basal level of rtTA expressed in our autoregulatory vector is not sufficient to avoid an immune response to rtTA.

Our results therefore suggest that danger signals associated with the injection of a viral vector are not essential for the development of an immune response. However, since our autoregulatory vector has, by definition, a low level of basal expression, we therefore cannot completely exclude the possibility that the immune system is primed at the moment of injection.

Further modifications are required to translate this system into clinical applications.

## Materials and Methods

### Plasmid construction

The constitutive rat Epo lentiviral vector was constructed by cloning rat Epo as an EcoR1 fragment in prrlcpptCMVPRE[27].

An expression plasmid containing the rtTA variant V16 (V9I F67S R171K), rtTA4, was digested with XbaI and XmaI and subcloned into a XbaI and XmaI digest of pRRLcpptMCSIRESrtTA3LTRTetO to make pRRLcpptMCSIRESrtTA4LTRTetO and was verified by sequencing. An expression plasmid for d2eGFP and pRRLcpptMCSIRESrtTA4LTRTetO were digested with BamHI and EcoRI and the resulting d2eGFP fragment was cloned into pRRLcpptMCSIRESrtTA4LTRTetO to make pRRLcpptd2eGFPIRESrtTA4LTRTetO. A BamHI and EcoRV digest was performed to liberate a fragment containing d2eGFPIRESrtTA4 and was subcloned into a BamHI and EcoRV digest of pBSK to make pBSK d2eGFPIRESrtTA4. An AgeI and XhoI digest was performed on pBSK d2eGFPIRESrtTA4 and TREAutoR3 and the new fragment d2eGFPIRESrtTA4 was cloned into the TREAutoR3 backbone to make TREAutoR4. The gene coding for rat erythropoietin was recovered by an EcoRI digest of the pRRLcpptCMVEpo plasmid cloned into the backbone of an EcoRI digest of TREAutoR4 to make TREAutoR4rEPO.

### Cell lines and culturing

Human embryonic kidney (HEK) 293T, HeLa, and H9c2 rat DB1X heart myoblasts were originally obtained from ATCC (http://www.atcc.org/), cultured in standard DMEM supplemented with 10% fetal bovine serum, 100 U/ml penicillin, 100 µg/ml streptomycin, 2 mM glutamine at 37°C in 10% CO_2_.

### Lentiviral vector preparation

Lentiviral vectors were produced as previously reported [27]. Briefly, HEK 293T cells were transiently transfected by calcium phosphate precipitation using a third generation lentiviral vector system [28], [29]. Twenty-four hours following transfection, fresh media supplemented with 25 mM HEPES buffer pH 7.4 was added. Virus containing supernatent was harvested 48 and 72 hours following transfected, filtered through 0.45 µm Millipore filters and concentrated by ultra centrifugation using a Beckman SW-28 rotor 20,000 RPM for 2 hours at 4°C. Viral pellets were resuspended in PBS and frozen at −80°C.

### Viral transduction

The p24 antigen content of concentrated TREAutoR4rEpo lentivirus was determined using a commercial ELISA kit from Perkin Elmer (NEK050A). H9C2 cells were seeded out at 1×10^5^ per well and were transduced for four hours in the presence of 10 µg/ml DEAE Dextran. Gene expression was induced by the addition of 10 ng/ml doxycycline (Sigma). Epo secretion into media was determined using a commercial ELISA kit from R&D Systems (DEP00). Viral titers of LVpgkGFP were determined by serial dilution on HeLa cells. GFP expression in transduced HeLa cells was determined by flow cytometry 72 hours following transduction.

### Ethics statement

All animal experiments were performed approved by the Animal Ethical Committee at the Academic Medical Center of Amsterdam.

### Intramuscular injection of CMV rEPO, TREAutoR4rEPO and LVpgkGFP

Male Wistar rats weighing approximately 300 g were anesthetized by isoflurane gas inhalation. The right hind leg was injected with a total of 100 µl of CMVrEpo, TREAutoR4rEPO virus (0.4 µg p24) or 100 µl of LVpgkGFP (0.4 µg p24, corresponding to1×10^7^ HeLa transducing units) in three separate injections.

### Doxycycline administration, blood collection, and analysis

Drinking water was prepared containing 200 ug/ml doxycycline, 1% sucrose pH 6.0. Blood was collected every two weeks via the tail vein under isoflurane gas anaesthesia. Hematocrit levels were determined using a glass capillary following standard protocols. Plasma was frozen at −20°C for determining rtTA antibody titers.

### ELISA for rat antibodies to rtTA

RtTA was expressed in 293T cells by calcium phosphate coprecipitation. Expression of rtTA was confirmed by western blotting. ELISA plates (Nunc) were coated overnight with 5 ug cellular lysate (rtTA or control) per well in 50 mM carbonate buffer pH 9.6. The wells were blocked with 1% gelatine in PBS, washed and incubated with serial dilutions of rat plasma collected at 2, 6, and 10 weeks following virus injection. After washing, rat immunoglobins were detected with anti-rat IgG peroxidase (Nordic) and o-phenylenediamine tablets (Sigma).

ELISA's were always performed in duplicate with the same samples applied to rtTA and control (293T cell lysate). To correct for a specific binding, the absorbance of the control plate was subtracted from rtTA coated plate. Rat plasma was considered to be positive for rtTA antibodies when the absorption of samples diluted 800 times was above the background.

### Immunostaining

*In vivo* formaldehyde fixed and sucrose embedded muscle tissue was snap frozen in liquid nitrogen. Sections of 7 µm were prepared and kept at −20°C prior to use. Sections were allowed to equilibrate at room temperature and washed three times in PBS for 5 minutes each. Subsequently sections were incubated for hour with a 1∶100 dilution of mouse anti rat CD45 (MCA43R AbD Serotec) in PBS/0.05% Tween-20/5% Normal goat serum/5% Rat plasma. Then sections were washed three times, five minutes each in PBS/0.05% Tween-20. A 1∶500 dilution of goat anti mouse IgG Alexa 594 in PBS/0.05% Tween-20/5% Normal goat serum/5% Rat plasma was performed for one hour followed by three wash steps in PBS five minutes each. Sections were mounted with Vectashield with DAPI (Vector laboratories H-1200). Images were captured at (200×) magnification using a fluorescent microscope (Leica DMRA2).

### SDS-PAGE and Western blotting

H9c2 cell lysates (50 µg) were separated on 10% SDS-PAGE and blotted onto nitrocellulose using the Bio-Rad Miniprotean III system. An antibody directed against the rtTA (TET02 MoBiTec) was used at a 1∶1000 dilution. An antibody directed against actin was purchased from NeoMarkers (Ab-5) and used at a dilution of 1∶1000. A 1∶1000 dilution of goat anti mouse HRP (Bio-Rad 170–6516) was used as the secondary antibody. Detection of reactive bands on western blot was performed using the Lumi-Light Western Blot Substrate (Roche 12 015 200 001) and blots were analyzed using a Lumi Imager F1 and Lumi Analyst 3.1 software (Roche).

### Statistical analysis

Statistical analysis was performed using SPSS 11.0 software using the Mann-Whitney U test. Values were determined to be significantly different with p<0.05.
